# The synergistic effect of internet-based health communication on health promotion: the chain-mediating effect of health literacy and health education

**DOI:** 10.3389/fpubh.2026.1934669

**Published:** 2026-10-12

**Authors:** Xuezhen Chen, Ziqi Zhang

**Affiliations:** School of Marxism, Wenzhou Polytechnic University, Wenzhou, China

**Keywords:** artificial intelligence, chain-mediating approach, health education, health literacy, health promotion, internet-based health communication, MOOCs, SPOC

## Abstract

Next-generation internet technologies, represented by artificial intelligence, are reshaping the nationwide health information distribution system; this study examines the synergistic benefit effect of online health communication on health promotion. The analysis utilizes 5,333 data points from the 2023 China General Social Survey (CGSS), which employed a nationally stratified, multi-stage sampling design. Using the Hayes PROCESS Model 6 serial chain mediation model, 2,000 bias-correction Bootstrap samples were generated to conduct baseline OLS regression, mediation effect decomposition, multidimensional heterogeneity analysis, and an IPW inverse probability weighting robustness test. The baseline OLS regression reveals a significant positive overall association between online health communication and health promotion. The chain mediation model identified three indirect pathways: a single health literacy mediation pathway, a single digital health education mediation pathway, and a core serial synergistic pathway (“Online Health Communication → Health Literacy → Digital Health Education → Health Promotion”); the confidence intervals for all three pathways do not include zero. The heterogeneity analysis uncovered the distribution pattern of the digital health gap: the serial chain effect is slightly higher in urban areas than in rural areas, and slightly higher among women than among men (though the intergroup difference is not statistically significant); the strongest effect is observed among the middle-aged population aged 31–60 years, while the serial transmission chain tends to weaken among groups with lower educational attainment. Multiple robustness tests, including IPW inverse probability weighting, support the reliability of the core findings. The positive association between online health communication and residents’ health promotion can be explained through the independent and serial mediation statistical pathways involving health literacy and digital health education; this observation reveals a “progressive synergy” relationship among the three factors.

## 1 Introduction

### 1.1 Conceptual explanation

The concept elucidates the profound application of internet technology in health communication, which has evolved from unidirectional information dissemination to a core driver reshaping the production, distribution, and consumption of health knowledge. “Internet-based health communication” refers to the process of conveying health knowledge to the public, influencing health perceptions, and guiding health behaviors through various channels—including search engines, social media, health apps, and generative artificial intelligence (AI)—utilizing diverse technological platforms such as internet-based systems encompassing search engines, social media, health applications, and AI-powered tools (1, 2). Multiple systematic reviews have demonstrated that AI-powered health dialogue systems show potential for intervention in real-world clinical and behavioral settings—particularly in promoting healthy lifestyles (3, 4); however, comprehensive national surveys targeting the general population still lack standardized measures to assess public usage behaviors regarding AI-based health tools. Eysenbach’s concept of “apomediation” provides a crucial theoretical framework for understanding how AI replaces traditional intermediaries by directly connecting health information with the public (5). A recent survey reveals that 17% of U.S. adults consult AI chatbots about health issues at least monthly, with this figure rising to 25% among those under 30 (3), demonstrating how internet-based health communication has evolved from a cutting-edge technology into mainstream practice.

Health literacy is defined as an individual’s ability to acquire, understand, evaluate, and apply health information to maintain or improve their own health (6). Sørensen et al. (7) developed a comprehensive model of health literacy through systematic literature review, delineating this concept into four core dimensions—acquisition, understanding, evaluation, and application—that span three key areas: healthcare, disease prevention, and health promotion; this model has served as the theoretical foundation for the European Health Literacy Survey (HLS-EU) (8). A systematic review by Stellefson et al. (9) revealed that college students generally exhibit low levels of e-health literacy, underscoring the need to integrate e-health education into university curricula. With the increasing prevalence of AI-generated health content, health literacy has evolved from a traditional concept to a multidimensional framework encompassing algorithmic awareness, information authenticity assessment, and critical evaluation skills (10). Research by Mackert et al. further confirms that health literacy levels directly influence individuals’ willingness to adopt and proficiency in using health information technologies (11).

Digital health education utilizes online educational platforms as its foundation, achieving deep internalization of health knowledge and long-term behavioral transformation through systematic and structured curriculum design (12). As core online teaching models in digital health education, MOOCs and SPOCs leverage internet technologies to overcome the temporal and spatial barriers inherent in traditional health literacy dissemination and education. Through differentiated instructional formats, audience targeting, and teaching approaches, they cater to diverse needs—including public health education, campus health instruction, and occupational health training—serving as crucial pillars for advancing the digitalization, scaling, and refinement of health education. Unlike fragmented health information consumption, digital health education emphasizes comprehensive knowledge systems, continuous learning, and interactive educational processes. This study posits that internet-based health communication, health literacy, and digital health education are not isolated entities but form a complete communication chain encompassing “information exposure → cognitive filtering → systemic internalization → behavioral transformation.” Notably, health literacy and digital health education exhibit a sequential relationship characterized by “screening before learning,” which forms the theoretical basis for constructing a serial chain-mediating model in this research.

The CGSS2023 is a comprehensive social survey that does not include dedicated survey items specifically designed to measure the use of generative AI-based health tools, algorithmic literacy, or participation in MOOC/SPOC health-related courses. The literature pertaining to generative artificial intelligence, chatbots, and algorithmic intermediaries is cited in this paper to establish the technological context within which this study is conducted; the empirical analysis utilizes proxy variables constructed from the existing items in the CGSS. While the selected survey items partially align theoretically with the core constructs, they do not encompass all dimensions of these constructs, resulting in certain measurement gaps; these mapping relationships and limitations will be elaborated upon in the Research Design section.

### 1.2 Literature review and research gaps

While existing studies have yielded preliminary achievements in the field of human-machine collaborative health communication, three significant gaps remain.

First, the limitations of sample representativeness. Most empirical studies published in current journals rely on convenient samples such as hospital patients and college students to conduct short-term human-computer interaction experiments (1, 3). While these studies exhibit high internal validity, their external generalizability is limited, making it difficult to reflect the heterogeneous characteristics of urban and rural residents nationwide. Domestic digital health literature based on national comprehensive surveys like CGSS and CFPS only examines the simple correlation between online information usage and health behaviors (13) without integrating internet dissemination characteristics with systematic online learning into a unified analytical framework (10). The systematic review by Chesser et al. highlights that the digital divide is particularly pronounced among vulnerable populations, yet existing literature has paid insufficient attention to this issue (14). Wu et al. note that China’s online health education faces dual challenges of the digital divide and the spread of unverified information (15).

Second, the theoretical limitations of the mediation model framework. Most similar studies employ parallel mediation models, treating variables such as health literacy and online learning as independent mediators in parallel, while overlooking the potential sequential relationship between them. Zhao et al.’s methodological research has long demonstrated that when there is a theoretically predetermined sequence among multiple mediating variables, the serial chain-mediating model provides a more accurate depiction of causal transmission mechanisms than the parallel mediation model (16). However, in the field of internet-based health communication, systematic investigations examining the complete chain of “information acquisition → cognitive filtering → systematic learning → behavioral transformation” remain scarce.

Third, there is a lack of a localized stratification perspective. Existing research on foreign-language chain-mediating effects primarily relies on homogeneous social samples from Europe and America, with limited attention paid to how structural factors such as urban–rural household registration and educational stratification in developing countries modulate or disrupt these transmission chains (14). Meanwhile, studies on MOOCS and SPOC health education focus mainly on continuing education for medical students, with few quantitative analyses addressing the implementation of lifelong learning mechanisms for general urban and rural residents (12).

### 1.3 Research questions

Given the aforementioned literature gaps, the contributions of this study are primarily reflected in the following aspects: utilizing the CGSS 2023 national stratified representative sample (10) to examine the chain-mediating pathway of “internet dissemination → health literacy → digital health education → health promotion behaviors”; simultaneously conducting multidimensional heterogeneity analysis across four dimensions—urban/rural, gender, age, and educational attainment—and employing inverse probability weighting (IPW) methods for robustness testing. The aforementioned study aims to examine the linear statistical correlation model of “Internet dissemination → Health Literacy → Digital Health Education → Health Promotion Behaviors,” providing a reference and empirical support for the development of tiered health education policies within the framework of the “Healthy China 2030” initiative.

## 2 Theoretical framework and research hypotheses

### 2.1 Direct effects of internet-based health communication on health promotion

The Human-AI Synergy Theory in Health Ecology posits that artificial intelligence is not merely a technical tool but a “cognitive partner” that co-evolves with human cognition (2). In the field of internet-based health communication, AI has significantly lowered the barrier to public access to health information through social media recommendation algorithms, intelligent Q&A systems, and personalized content generation. A case study conducted by Kilic et al. at Sri Lankan public health institutions demonstrated that AI-generated images achieved 30–40% higher engagement rates on health-focused social media platforms compared to non-AI content (1). Romero-Rodriguez and Castillo-Abdul further argue that generative AI enables contextualized health information narratives that reduce cognitive processing demands and enhance message persuasiveness (2). A randomized controlled trial by Bickmore et al. confirmed that automated health coaching significantly improves physical exercise participation among older adults populations (17). Based on these theoretical frameworks and empirical evidence, this study proposes the following conclusions:

*Research Hypothesis 1*: Internet-based health communication exerts a significant positive overall effect on promoting residents’ health.

### 2.2 The single mediating role of health literacy

The role of health literacy as a key intermediary between online health communication and health promotion is explained by the mechanism of “cognitive filtering.” Empirical studies by Berkman et al. demonstrate that the impact of information acquisition on health behaviors predominantly operates through the pathway of health literacy, with individuals possessing higher health literacy being better able to extract meaningful information from information overload and translate it into actionable strategies (18). Bandura’s social cognitive theory provides a macro-level theoretical framework for understanding this mechanism: individual health behaviors are influenced by a complex interplay of self-efficacy, outcome expectations, and environmental factors, with health literacy serving as the core capacity that modulates this interaction (19). Paasche-Orlow and Wolf proposed a causal pathway model through which health literacy affects health outcomes, encompassing three channels: healthcare accessibility, physician-patient interactions, and self-management (20). A systematic review by Berkman et al. further confirms that low health literacy is significantly associated with higher hospitalization rates, lower vaccination rates, and poorer medication adherence (18). Based on these findings, the following conclusion is drawn:

*Research Hypothesis 2*: Health literacy plays a significant independent mediating role between internet-based health communication and health promotion.

### 2.3 The chain-mediating effect of digital health education

The Lifelong Online Education Theory emphasizes that fragmented exposure to knowledge cannot replace a systematic educational process (12). In the field of health promotion, online health communication primarily delivers fragmented, context-specific knowledge points that may trigger brief cognitive awareness but fail to establish stable cognitive schemas or behavioral habits. True transformation into healthy behaviors requires structured, systematic health education to integrate and deeply internalize knowledge. More crucially, digital health education does not serve as a parallel intermediary to health literacy; rather, it constitutes a subsequent phase following the exertion of health literacy—the public must first possess sufficient health literacy to identify and filter effective information generated by AI before actively engaging in systematic online courses.

Li et al. (12) conducted a quasi-experimental study targeting nursing professionals to demonstrate the role of systematic education under the SPOC model in enhancing health literacy; this finding provides empirical support for the theoretical hypothesis of this study—that “systematic learning facilitates the transformation of health behaviors.” It is important to note that the CGSS questionnaire did not directly measure the usage behavior of general residents toward MOOC or SPOC platforms; instead, M2 only captured residents’ general learning tendencies and does not represent a direct observation of participation in digital health-related courses.

Research by Neter and Brainin demonstrates that electronic health literacy significantly widens the digital divide, with low-literacy groups exhibiting clear disadvantages in accessing, understanding, and applying electronic health information (21). The Theory of Change Model (TTM) developed by Prochaska and Velicer provides an analytical framework for understanding different stages of behavior change: internet-based health communication primarily influences the “contemplation phase,” health literacy facilitates progression to the “preparation phase,” while systematic education ultimately drives behavioral transformation during the “action phase” and “maintenance phase” (22). From this, it follows that:

*Research Hypothesis 3*: A significant serial chain-mediating pathway exists— “Internet-based health communication → health literacy → digital health education → health promotion” —where these three elements form a progressive, synergistic mechanism.

It is important to note that the H1H3 hypothesis represents a causal mechanism assumption derived from theoretical reasoning. However, this study employs cross-sectional data for testing; such an approach can only determine whether the statistical association patterns among the variables align with the theoretical expectations, but it cannot ascertain the temporal sequence of the variables nor directly establish a causal relationship. Therefore, relevant causal inferences require further validation through longitudinal panel data analyses or intervention experiments.

## 3 Research design

### 3.1 Data sources and sample selection

The data for this study were obtained from the 2023 China General Social Survey (CGSS). This project was conducted by the China Survey and Data Center at Renmin University of China, employing a dual-track, multi-stage stratified probability sampling method combining household interviews and telephone surveys. It covered all 31 provinces, autonomous regions, and municipalities directly under the central government nationwide, yielding 11,326 valid questionnaires in total, which included 437 observed variables and comprehensively addressed all core modules such as internet usage, self-assessed health, online learning, and demographic and socioeconomic characteristics (10). The publicly available CGSS2023 Open-Access Dataset provides individual sampling weights; however, it does not disclose the identifiers for Primary Sampling Units (PSUs) or strata units. As a result, ordinary users cannot fully replicate the cluster-stratification adjustment required for complex sampling designs.

Sample cleaning adheres to the following criteria: observations with missing core variables or invalid responses (e.g., “unknown/ refused to answer”) are excluded; only adult respondents aged 18 or older are retained. The missing rate for core measurement items ranges from 38 to 51% (for item A313 “Learning and Professional Development,” the missing rate reaches up to 50.8%); the missing rate for control variables is all below 5%. After removing incomplete cases, the final valid analysis sample consists of N = 5,333. Ethical Note: The CGSS Public Secondary Database has been approved by the Institutional Ethics Review Board of Renmin University of China; all respondents’ personal information has been fully desensitized and anonymized.

This study performed data cleaning using the raw valid questionnaires from the public release of the CGSS2023 survey, sequentially excluding the following types of samples: ① samples with missing values for core variables (Internet usage, composite health literacy items, leisure learning, physical activity, or demographic control variables); ② samples whose total response time fell below the valid response threshold or who exhibited clear signs of arbitrary responding; ③ samples in which key items contained logical inconsistencies (e.g., age did not match the birth year, or income values exceeded the reasonable range). After this cleaning process, a final valid sample of 5,333 observations was included in the analysis. Sample attrition may introduce selection bias: missing values are predominantly concentrated among individuals with lower educational attainment, older age groups, or those living in rural areas, which could lead to an underestimation of the proportion of these groups within the final sample. To address this issue, this study employed the inverse probability weighting (IPW) method in the robustness checks—using demographic characteristics to predict the probability of sample inclusion and then weighting the model accordingly—thereby ensuring that the core chain-mediation conclusions remained robust and reducing the estimation bias arising from sample attrition to some extent. However, due to the limitations of the secondary data, it is not possible to completely eliminate unobserved selection bias.

### 3.2 Measurement indicators

#### 3.2.1 Indicator concept and specific composition

Independent Variable: Internet-based health communication (X). This study employs internet usage intensity as a proxy variable for internet-based health communication (10). The CGSS 2023 questionnaire includes two items: Item A285 asks respondents about “internet usage frequency,” with options ranging from 1 (“never”), 2 (“rarely”), 3 (“sometimes”), 4 (“often”), to 5 (“very frequently”); Item A29 asks about “primary information source,” with choices including newspapers, magazines, radio, television, and the internet. In this study, Item A29 was coded as a binary variable (Internet = 1; other = 0), and both items were standardized before factor analysis was conducted to extract factor scores, which were then synthesized into an internet-based health communication index. Compared to using mean values, factor scores more accurately reflect the distinct contributions of each item to the latent variable. The rationale behind constructing this proxy variable is that the dissemination of AI-driven health information relies on internet infrastructure; thus, a higher level of internet usage intensity indicates that individuals are more likely to be exposed to the ecosystem of internet-based (including AI-generated) health information. However, this indicator cannot confirm whether respondents have actually browsed AI-generated health-related content.

First-order mediator variable: Health literacy (M1). The measurement of health literacy draws on the multidimensional assessment methodology proposed by van der Vaart and Drossaert (23) for digital health literacy, as well as the integrated model of health literacy developed by Sørensen et al. (7). Based on items from the CGSS 2023 questionnaire, this study synthesized a health literacy index across three dimensions. The first dimension measures linguistic competence, utilizing items A49 (“Mandarin listening ability” [1 = completely unable to understand; 5 = very good]) and A50 (“Mandarin speaking ability” [1 = completely unable to speak; 5 = very good]), reflecting an individual’s language foundation for accessing and understanding health information. The second dimension assesses cognitive baseline levels through item A7a (“highest educational attainment” [1 = no education; 14 = postgraduate level or above]), as educational attainment has been widely recognized as a core predictor of health literacy (18). The third dimension evaluates proactive reading behavior via item A3004 (“frequency of reading newspapers/magazines during leisure time” [original coding: 1 = daily; reverse-coded to 1 = never; 5 = daily]), indicating an individual’s initiative in obtaining health information through reading. The health literacy index was calculated as the mean of the standardized scores from these four items, with a Cronbach’s α coefficient of 0.737, meeting acceptable reliability standards for social science research.

Second-order mediator variable: Digital Health Education (M2). Item A313 in the CGSS 2023 questionnaire asks about “the frequency of learning during leisure time,” with options ranging from 1 (never) to 5 (very frequently), reflecting respondents’ level of active engagement in systematic knowledge acquisition within informal educational settings. In the digital era, learning activities increasingly occur through online education platforms such as MOOCs and SPOCs; thus, this item serves as an effective proxy for measuring the frequency of participation in digital health education (12). This study standardized this item and used it as the measurement indicator for M2.

Dependent variable: Health promotion (Y). The frequency of physical exercise was used as the measurement indicator for residents’ health promotion level (10, 24). Item A3009 in CGSS 2023 asks about “frequency of physical exercise,” with a raw code scale ranging from 1 (daily) to 5 (never). In this study, the scale was reversed to 1 = never, 2 = once per week or less, 3 = several times per week, 4 = approximately weekly, and 5 = daily, where higher scores indicate greater exercise frequency and better health promotion levels.

Control variables: This study includes a total of 13 control variables across three levels. Individual-level control variables include: gender (A2; male = 1; female = 0), age (calculated from the birth year in A3a:2023 − birth year), ethnicity (A4; Han = 1; other = 0), political affiliation (A10; Party member = 1; other = 0), and self-rated health status (A15; 1 = very unhealthy to 5 = very healthy). Educational attainment is the core compositional dimension of the health literacy mediator M1 and constitutes an integral part of this mediator variable. To mitigate post-treatment bias in mediation analysis, this study does not repeatedly include educational attainment as a control variable in the model’s set of control variables. If the constituent elements of the mediator variable were simultaneously treated as covariates for control, this would effectively control for only a portion of the mediation chain, thereby distorting the estimation of the path coefficients for the X → M₁ and M₁ → Y pathways and introducing model specification bias. In this study, educational attainment is used solely to construct the M1 health literacy index; in the subsequent heterogeneity analysis, educational attainment will be employed as a grouping moderator variable for subsample analysis. However, conducting a grouping analysis is not equivalent to including this variable in the regression’s set of control variables. Family-level control variables include: urban/rural household registration status (generated from A25a; urban = 1, rural = 0), annual household income (A62, transformed using the natural logarithm), number of properties owned (A65), marital status (A69; married = 1, otherwise = 0), and household size (A1). Social trust-level control variables include: general social trust (A33; 1 = very distrustful to 5 = very trusting), trust in doctors (C17f; 1 = completely distrustful to 4 = very trusting), and participation in medical insurance (A611; insured = 1, uninsured = 0).

The selection of these control variables was based on Paasche-Orlow and Wolf’s (20) causal pathway model of health literacy and Berkman et al.’ s (2011) systematic review on how health literacy influences health behaviors.

#### 3.2.2 Theoretical mapping of observation indicators and measurement limitations

This study utilizes data from the CGSS2023 Comprehensive Social Survey; however, this questionnaire is not a dedicated digital health survey, and it does not contain standardized, specialized scales for measuring health literacy or digital health education. As a result, all observed variables selected for this study serve as proxy indicators for the respective theoretical constructs: the selected items capture certain underlying dimensions of these constructs but cannot fully encompass their entire conceptual scope, indicating inherent measurement limitations. The theoretical mapping relationships among the variables are explained as follows:

1. Independent variable: Internet health communication (X). The theoretical construct refers to the process of disseminating health-related information over the Internet, encompassing search engines, social media platforms, and generative AI. This study uses a composite index derived from internet usage frequency and whether the Internet serves as the primary source of information. From a theoretical perspective, high-frequency Internet use and reliance on the Internet as the primary information channel represent necessary prerequisites for individuals coming into contact with Internet-based health information (including health content generated by AI); this index reflects the potential exposure of individuals to the Internet health information ecosystem. However, this measure cannot distinguish the specific topics of the information viewed by respondents, nor can it directly confirm whether respondents have actually encountered health-related content or AI-generated health information.
2. First-order mediating variable: Health Literacy (M1). According to the Integrated Health Literacy Model developed by Sørensen et al. (7), health literacy encompasses four core dimensions: acquiring, understanding, evaluating, and applying health-related information. This study constructs a composite Health Literacy Index by combining Mandarin language proficiency, educational attainment, and daily reading behavior: Mandarin listening and speaking skills serve as the linguistic foundation for individuals ‘ability to comprehend oral and textual health-related information; educational attainment provides the cognitive basis for individuals’ information processing and logical reasoning; while leisure reading habits reflect individuals’ behavioral tendencies toward actively seeking and receiving various types of information. Together, these three dimensions correspond to the underlying components of health literacy—namely “understanding information, basic cognition, and active information acquisition.” However, due to limitations imposed by the survey instrument, this indicator does not cover higher-order, specialized dimensions of health literacy—such as discerning the authenticity of health information, identifying algorithmic biases, or conducting specialized assessments of health knowledge—and does not align with internationally recognized standardized scales (e.g., HLS-EU or eHEALS); thus, it represents an incomplete proxy measure. The reliability and validity analyses conducted in this study demonstrate that this composite indicator exhibits acceptable reliability and a unidimensional structure, providing methodological support for its use as a proxy measure.
3. Second-order mediating variable: Digital Health Education (M2). This theoretical construct refers to systematic health education initiatives delivered through digital platforms. In this study, the single-item measure “frequency of learning and knowledge enrichment during leisure time” is used as a proxy variable. This item reflects an individual’s subjective inclination to engage in systematic and continuous knowledge acquisition; in the current context of widespread digital media adoption, a significant portion of residents’ leisure learning activities is conducted via online digital channels, providing a solid practical basis for using this item as a proxy for digital health education behavior tendencies. However, as this is a generic learning-related item, it cannot distinguish whether the learning content pertains to health-related topics or whether the learning takes place on MOOC/SPOC online platforms or through offline printed materials; consequently, the indicator contains some measurement noise.
4. Dependent variable: Health Promotion (Y). Health promotion is a broad concept encompassing cognitive, psychological, and multidimensional behavioral dimensions. The frequency of physical exercise serves as a key, observable, and objective behavioral indicator within health promotion behaviors; it has been used as a proxy outcome variable for health promotion in numerous previous empirical studies on digital health. However, a single physical exercise metric cannot capture other health promotion behaviors—such as diet, sleep, or adherence to medical care—and thus only represents a partial dimension of health promotion.

In conclusion, this study’s empirical model empirically tests the sequential transmission pathway: Internet information exposure potential → foundational health literacy competencies → general systematic learning tendency → physical exercise behavior; based on these statistical findings, it then infers the underlying theoretical mechanisms within the context of Internet-based health communication. The dimensionality gap arising from proxy indicators represents an unavoidable constraint inherent in comprehensive secondary social survey data, which limits the precision of the research inferences.

#### 3.2.3 Reliability and validity analysis

Regarding reliability, internal consistency tests were conducted for the composite variable comprising multiple items. For M1 (Health Literacy), the Cronbach’s α for the four items was 0.737; after item deletion, the *α*-values ranged from 0.633 to 0.755, with total item-relatedness between 0.441 and 0.584 and inter-item correlation coefficients ranging from 0.240 to 0.639 (all *p* < 0.001), meeting the acceptable reliability standards for social science research. For X (Internet-based health communication), comprising two items (frequency of internet use and primary information sources), the Cronbach’s α was 0.598, the Spearman-Brown’s split-half reliability was 0.858, and the inter-item correlation coefficient was *r* = 0.751 (*p* < 0.001), indicating satisfactory split-half reliability. Both M2 (Digital Health Education) and Y (Health Promotion) were measured as single-item constructs, thus requiring no calculation of internal consistency reliability.

Regarding validity, exploratory factor analysis (EFA) was employed for evaluation. For the M1 questionnaire with four items, the KMO = 0.677 and the Bartlett’s test of sphericity yielded a *χ*^2^ = 5504.5 (*p* < 0.0001), indicating that the data are suitable for factor analysis. Using the Kaiser criterion (where the eigenvalue is >1), one common factor was extracted (with an eigenvalue of 2.255). According to the eigenvalue method, this factor accounts for 56.4% of the total variance (versus 43.3% when calculated using the EFA communality measure; since these two measures correspond to different variance benchmarks, they cannot be directly compared). The factor loadings for each item are as follows: Mandarin listening – 0.707; Mandarin speaking – 0.784; Educational attainment – 0.655; Reading frequency – 0.433; thus, the unidimensional assumption for M1 is largely satisfied. For the X questionnaire with two items, the KMO = 0.500; one common factor was extracted, with all factor loadings equal to 0.866, indicating that the two items contribute highly consistently to the same construct. The full set of six items from both X and M1 was subsequently included in an exploratory factor analysis (EFA), yielding a KMO = 0.717 and a Bartlett’s *χ*^2^ = 11353.3 (*p* < 0.0001). Two common factors with eigenvalues >1 were extracted (cumulative variance explained = 67.3%). After rotation, the factor loadings exhibited a clear distribution pattern: for Factor 1, the four M1 items all displayed high loadings (0.401–0.767); for Factor 2, the two X items all displayed high loadings (0.809–0.858). With the exception of educational attainment, which had a slightly higher loading on Factor 2 (0.308), all other cross-loadings were below 0.3, indicating that X and M1 possess good discriminant validity.

The aforementioned validity and reliability analysis provides a solid methodological foundation for the subsequent variable synthesis in the chain-mediating model.

#### 3.2.4 Collinearity diagnosis

In this study, the Variance Inflation Factor (VIF) test was conducted for all variables included in the regression model (including the composite dimension of the mediator variable M1 and all control variables). The results indicate that the VIF values for all variables are below 5, and the average VIF falls within the acceptable range for social science research; this suggests that the model does not suffer from severe multicollinearity issues, and the estimated regression coefficients are stable. It should be noted that, as a core component dimension of the mediator variable M1, educational attainment exhibits a theoretically moderate correlation with control variables such as family economic status and urban/rural household registration status; furthermore, there are certain interrelationships among the components of M1—namely, Mandarin proficiency, educational attainment, and reading habits. This is an inevitable feature when constructing composite indicators using secondary data from comprehensive social surveys; it may slightly inflate standard errors or reduce the power of statistical tests, representing an inherent limitation at the measurement level of this study.

### 3.3 Statistical model specification

#### 3.3.1 Three-stage empirical analysis strategy

This study employs a three-stage empirical analysis strategy.

The first stage involves the benchmark effect test. Three stepwise OLS regression models were constructed, sequentially incorporating independent variables, individual control variables, and full control variables, to examine the overall effect of internet-based health communication on health promotion.

The second stage involves a chain-mediated moderation test. The Hayes PROCESS Model 6 serial chain-mediated moderation model (25) was employed, with 2,000 bootstrap replicates performed for bias correction; the total effect was then decomposed into a direct effect and an indirect effect. The indirect effects were further broken down into three pathways: X → M1 → Y (single health literacy pathway), X → M2 → Y (single digital health education pathway), and X → M1 → M2 → Y (core serial chain synergy pathway), with the contribution ratios of each pathway quantified (25).

The third stage involves multidimensional heterogeneity analysis and robustness testing. Chain-mediating models were estimated for subgroups across urban–rural differences, gender, age groups, and educational levels using the full sample, with intergroup coefficient differences compared via Bootstrap confidence intervals; IPW inverse probability weighting was employed to correct for potential sample self-selection bias (25). This pathway structure constitutes the theoretical conceptual framework of the study.

The serial chain-mediating model (PROCESS Model 6) developed in this study comprises three transmission chains and involves five core path coefficients.

In the first stage (X → M1), the effect coefficient of internet-based health communication on health literacy was a1 = 0.1660 (*p* < 0.001), indicating that exposure to internet health information significantly enhances residents’ health literacy.

In the second stage (X → M2, M1 → M2), internet-based health communication exhibited a direct effect on digital health education of a₂ = 0.0677 (*p* < 0.001), whereas health literacy demonstrated a progressive effect of d₂₁ = 0.4181 (*p* < 0.001) on digital health education—with the latter effect magnitude significantly exceeding the former. This indicates that health literacy serves as a critical prerequisite for driving individuals’ active participation in systematic online learning.

In the third stage (M1 → Y, M2 → Y), health literacy demonstrated a significant effect on health promotion (b1 = 0.1575, *p* < 0.001), while digital health education showed an even stronger effect (b2 = 0.2270, *p* < 0.001), indicating that systematic learning enhances behavioral change more effectively than general literacy levels. Additionally, the direct effect of online health communication on health promotion was modest (c = 0.0282, *p* > 0.05); this effect became insignificant after controlling for mediating variables, confirming that the independent variable’s impact on the dependent variable is entirely mediated by M1 and M2.

Based on the above path coefficients, the calculation results for the three indirect effects are as follows: IE1 (single health literacy pathway, X → M1 → Y) = 0.0262 (95% CI [0.0197, 0.0333]), accounting for 45.9% of the total indirect effect; IE2 (single digital health education pathway, X → M2 → Y) = 0.0152 (95% CI [0.0090, 0.0219]), accounting for 26.6%; IE3 (core chain-mediating pathway, X → M1 → M2 → Y) = 0.0157 (95% CI [0.0123, 0.0193]), accounting for 27.5%. The Bootstrap confidence intervals for all three indirect pathways exclude zero, indicating statistically significant effects. The chain-mediating model demonstrates an overall explanatory power of *R*^2^ = 0.1801 for health promotion.

#### 3.3.2 Explanation of complex sampling design and sampling weights

The CGSS employs a hierarchical, multi-stage probability-based complex sampling design. However, due to two constraints, the primary benchmark regression in this study and the Hayes PROCESS Model 6 chained mediation Bootstrap primary analysis did not directly incorporate sampling weights, cluster adjustment, or stratification adjustment. First, the Hayes PROCESS macro itself is not compatible with the “survey” module in R/Stata, making it impossible to directly invoke sampling weights within the chained mediation Bootstrap framework to perform mediation effect decomposition; currently, there are no established tools for implementing bias-correction Bootstrap methods for serial chained mediation under complex sampling designs. Second, the publicly available CGSS 2023 dataset does not provide identifiers for primary sampling units (PSUs) or stratification units, making it impossible to perform cluster-robust standard error adjustment. As an alternative robustness strategy, this study employs IPW (Inverse Probability Weighting) regression adjustment, constructing weights based on demographic characteristics to partially mitigate estimation biases arising from sample selection and sampling representativity; the core path coefficient obtained after IPW weighting remains consistent in direction and significance with that of the primary model. However, IPW is not equivalent to the official complex survey sampling weights provided by CGSS, and thus cannot fully replicate the original complex sampling design. The aforementioned tool and data constraints represent objective limitations inherent in the analysis of secondary publicly available data.

### 3.4 Statistical model formula

The total effect is the sum of the direct effect and the total indirect effects: Total Effect = c + Total IE. The standard errors and confidence intervals for each indirect effect were estimated using a bias-corrected Bootstrap method (B = 2000 repeated samples). Specifically, 2000 Bootstrap samples were drawn with replacement from the original sample; in each sample, all path coefficients of the three regression equations were reestimated and their product terms calculated, with 95% confidence intervals constructed at the 2.5th and 97.5th percentiles of the empirical distribution. If the interval does not contain zero, the indirect effect is statistically significant at the alpha = 0.05 level.

This study adopts PROCESS Model 6 proposed by Hayes (25) as the benchmark model to construct the following serial chain-mediating equation system. Let X represent internet-based health communication, M1 denote health literacy, M2 represent digital health education, Y indicate health promotion, and C denote the control variable vector. The path coefficients are jointly estimated using the following three regression equations:

$$M_{1}=\beta_{0}+a_{1}X+\gamma_{1}C+\varepsilon$$

$$M_{2}=\beta_{0}+a_{2}X+d_{21}M_{1}+\gamma_{2}C+\varepsilon_{2}$$

$$Y=\beta_{0}+c^{’}X+b_{1}M_{1}+b_{2}M_{2}+\gamma_{3}C+\varepsilon_{3}$$

Here, a₁ denotes the direct effect of X on M₁, a₂ denotes the direct effect of X on M₂, d₂₁ denotes the direct effect of M₁ on M₂, c represents the direct effect of X on Y after controlling for mediators, while b₁ and b₂ denote the direct effects of M₁ and M₂ on Y, respectively. Based on these path coefficients, the calculation formulas for the three indirect effects are as follows:

$$\mathrm{IE}₁=a_{1}.b_{1}\;(\text{Single Health Literacy Approach}:X\to M_{1}\to Y)$$

$$\begin{array}{l} IE_{2}=a_{2}.b_{2}\\ \;\;\;\;\;\;\;(A\;\text{single digital pathway for health education}:X\to M_{2}\to Y) \end{array}$$

$$\begin{array}{l} IE_{3}=a_{1}.d_{21}.b_{2}\;\\ \;\;\;\;\;(\text{Serial Chain Collaborative Path}:X\to M_{1}\to M_{2}\to Y) \end{array}$$

$$\text{Total}\;\mathrm{IE}=IE_{1}+IE_{2}+IE_{3}$$

Heterogeneity analysis employed a clustered Bootstrap strategy: the entire sample was divided into several subgroups based on urban/rural status, gender, age group, and educational level. The aforementioned PROCESS Model 6 was independently run in each subgroup, and the statistical significance of intergroup differences was assessed by comparing the overlap of Bootstrap confidence intervals between groups. Robustness testing utilized inverse probability weighted regression adjustment (IPWRA), where high/low exposure groups were defined using the median value of internet-based health communication as a threshold; propensity scores were estimated using a Logistic model to construct IPW weights, followed by weighted least squares regression analysis.

$$\text{Bootstrap}\;\mathrm{CI}(IE_{j})=[P_{2.5}({\hat{\beta }}_{j}*),P_{97.5}({\hat{\beta }}_{j}*)]$$

## 4 Empirical results

### 4.1 Descriptive statistics and correlation analysis

The effective sample size was *N* = 5,333. The sample composition was as follows: males accounted for 44.97% (2,398 individuals), females for 55.03% (2,935 individuals); mean age was 54.3 years (SD = 16.3), with an age range of 18 to 94 years; urban residents constituted 42.3%, while rural residents accounted for 57.7%; those with a bachelor’s degree or higher represented 9.8%. The mean value of the dependent variable Y (frequency of physical exercise) was 2.58 (SD = 1.62), indicating a moderately low level, which is consistent with the epidemiological findings on physical activity levels among Chinese adults reported by Tudor-Locke et al. (24). Analyses conducted by Tudor-Locke et al. (24) and Wei and Jia (10) using CGSS data also provided comparable descriptive references for this study.

Table 1 presents the descriptive statistics and Pearson correlation matrices for the core variables. All four core variables exhibit significant positive correlations pairwise (*p* < 0.001): the correlation coefficient between Internet-based health communication (X) and health promotion (Y) is 0.1607; between X and health literacy (M1) is 0.4390; between M1 and digital health education (M2) is 0.5690; and between M2 and Y is 0.3482. Notably, the correlation coefficient between M1 and M2 (*r* = 0.5690) is the highest among all pairs, preliminarily confirming a strong progressive relationship between them. The Cronbach’s *α* coefficient for the M1 composite indicator is 0.737, meeting the reliability standards accepted in social science research. Variance inflation factor (VIF) analysis shows that all independent variables have VIF values below 2.1 (the maximum value is 2.030), well below the commonly used threshold of 10, indicating no significant multicollinearity issues.

**Table 1:** Descriptive statistics and correlation matrices of core variables (*N* = 5,333).

Variable	M	SD	X	M1	M2	Y
Internet health comm. (X)	−0.1024	0.9599	1			
Cognitive ability (M1)	−0.1608	0.7036	0.4390	1		
Digital health edu. (M2)	0.0139	1.0035	0.3345	0.5690	1	
Health promotion (Y)	2.5828	1.6196	0.1607	0.3332	0.3482	1

The standardized Cronbach’s *α* coefficient for the M1 synthesis indicator (complete sample with four items, *N* = 5,322) is 0.737; the correlation coefficients among the variables are *p* < 0.001.

### 4.2 Benchmark regression results

Table 2 presents the results of a stepwise OLS regression analysis examining the role of online health communication in health promotion. In Model 1, which includes only the independent variable, the regression coefficient for online health communication is 0.161 (SE = 0.014, *p* < 0.001), with an *R*^2^ of 0.026. When Model 2 incorporates individual-level control variables—including gender, age, ethnicity, and political affiliation—the regression coefficient is 0.150 (SE = 0.017, *p* < 0.001), and the *R*^2^ increases to 0.048. In Model 3, which further includes household-level variables (logarithm of household income, number of properties, marital status, and household size) as well as social trust variables (general trust, trust in physicians, participation in health insurance, and self-rated health), the regression coefficient is rounded to 0.086 (SE = 0.017, *p* < 0.001); this value differs slightly from the original total effect *β* = 0.0855 reported in the chain mediation model—this discrepancy is merely due to rounding. This finding indicates that, after controlling for all potential confounding factors, there remains a significant positive statistical association between online health communication and health promotion; the estimated statistical effect size remains stable across different model specifications. This observation aligns with the theoretical expectations of Research Hypothesis H1.

**Table 2:** Benchmark OLS regression results (standardized coefficients *β*).

Variable	Model 1	Model 2	Model 3
Internet health comm. (X)	0.161*** (0.014)	0.150*** (0.017)	0.086*** (0.017)
Individual controls	No	Yes	Yes
Family/social controls	No	No	Yes
*R* ^2^	0.026	0.048	0.111
*N*	5,333	5,333	5,333

The values in parentheses represent HC3 heteroskedasticity-robust standard errors; ***p < 0.001; education level is used solely to construct the health literacy measure M1 and is not included as a control variable in this table’s regression analysis to avoid post-hoc bias. The coefficient of 0.086 in this table reflects the OLS regression results, which is the rounded value of the raw total effect *β* = 0.0855 obtained from the PROCESS chain-mediation model; since these two estimates are derived from two distinct software calculation procedures, there is a minor rounding difference at the third decimal place; however, the core model specification remains fully consistent.

### 4.3 Chain-mediating effect decomposition

The estimation results of the path coefficients for the chain-mediated model indicate that all path coefficients are statistically significant. The direct effects of X on M1 (a1 = 0.1660, *p* < 0.001), X on M2 (a2 = 0.0677, *p* < 0.001), M1 on M2 (*d* = 0.4181, *p* < 0.001), M1 on Y (b1 = 0.1575, *p* < 0.001), and M2 on Y (b2 = 0.2270, *p* < 0.001) all exhibit significant positive relationships. The Bootstrap 95% confidence interval for the direct effect (c’ = 0.0282) includes zero (*p* > 0.05), indicating that the impact of internet-based health communication on health promotion is mediated by intermediate variables, reflecting a predominantly indirect transmission pattern. The total explanatory power of the chain-mediating model for health promotion is R^2^ = 0.1801.

Table 3 presents the effect decomposition results based on 2,000 bootstrap samples with bias correction, which represent the core findings. The total effect is 0.0855 (95% CI [0.0528, 0.1178]), with the direct effect being statistically insignificant. The total indirect effect amounts to 0.0572 (95% CI [0.0474, 0.0675]), accounting for 66.9% of the total effect.

**Table 3:** Results of bootstrap chain-mediating analysis.

Effect type	Path	Coefficien *β*	95% bootstrap CI	Proportion of total indirect effect
Total effect	X → Y	0.0855	[0.0528, 0.1178]	—
Direct effect	X → Y (c’)	0.0282	[−0.0036, 0.0596]	N.S.
Total IE	IE1 + IE2 + IE3	0.0572	[0.0474, 0.0675]	100%
IE1	X → M1 → Y	0.0262	[0.0197, 0.0333]	45.9%
IE2	X → M2 → Y	0.0152	[0.0090, 0.0219]	26.6%
IE3 (Chain)	X → M1 → M2 → Y	0.0157	[0.0123, 0.0193]	27.5%

Bootstrap sampling was performed 2000 times; *** indicates that the 95% confidence interval does not include 0 (significant); N.S. indicates non-significant.

Specifically, the three indirect pathways exhibit the following characteristics: First, the effect size of the single health literacy-mediated pathway (X → M1 → Y) is 0.0262 (95% CI [0.0197, 0.0333]), accounting for 45.9% of the total indirect effect. Health literacy independently transforms internet-disseminated health information into behavior-promoting outcomes, confirming its core function as a “cognitive barrier.” Second, the effect size of the single digital health education-mediated pathway (X → M2 → Y) is 0.0152 (95% CI [0.0090, 0.0219]), representing 26.6% of the total indirect effect, indicating the independent driving role of systematic online learning in health behaviors. Third, and most notably, the chain-based synergistic pathway (X → M1 → M2 → Y) has an effect size of 0.0157 (95% CI [0.0123, 0.0193]), contributing 27.5% to the total indirect effect; the Bootstrap confidence interval does not include zero, this demonstrates that the three-tiered chain-based transmission mechanism involves an independent statistical mediating effect. Since the data are cross-sectional in nature, it is not possible to determine the temporal sequence of the variables; therefore, this chain merely represents the statistical association pattern among the variables. From a theoretical perspective, if this causal mechanism holds true, then either simply disseminating AI-driven health information or solely enhancing individual health literacy may be insufficient to maximize the effectiveness of health promotion efforts; instead, integrating systematic online learning is likely to unlock the potential for synergistic benefits.

Figure 1 presents a bar chart comparing the effect sizes and their 95% confidence intervals for the three indirect pathways. IE1 (X–M1–Y, single health literacy pathway) exhibits the highest effect size of 0.0262, accounting for 45.9% of the total indirect effects; its confidence interval [0.0197, 0.0333] is entirely within the positive range with a narrow width, indicating that health literacy serves as the most statistically robust and significant independent mediating pathway. IE2 (X–M2–Y, single digital health education pathway) has an effect size of 0.0152 (26.6%), with a confidence interval [0.0090, 0.0219] that is statistically significant but relatively wide, reflecting moderate variability across bootstrap samples. IE3 (X–M1–M2–Y, core serial chain-mediating pathway) demonstrates the most compact confidence interval [0.0123, 0.0193] with an effect size of 0.0157 (27.5%), highlighting strong consistency in the chain-mediating mechanism. The confidence intervals for all three pathways do not include 0 (with partial overlap between the intervals for IE2 and IE3), demonstrating that the three conduction mechanisms contribute statistically independently and complementarily.

**Figure 1:**
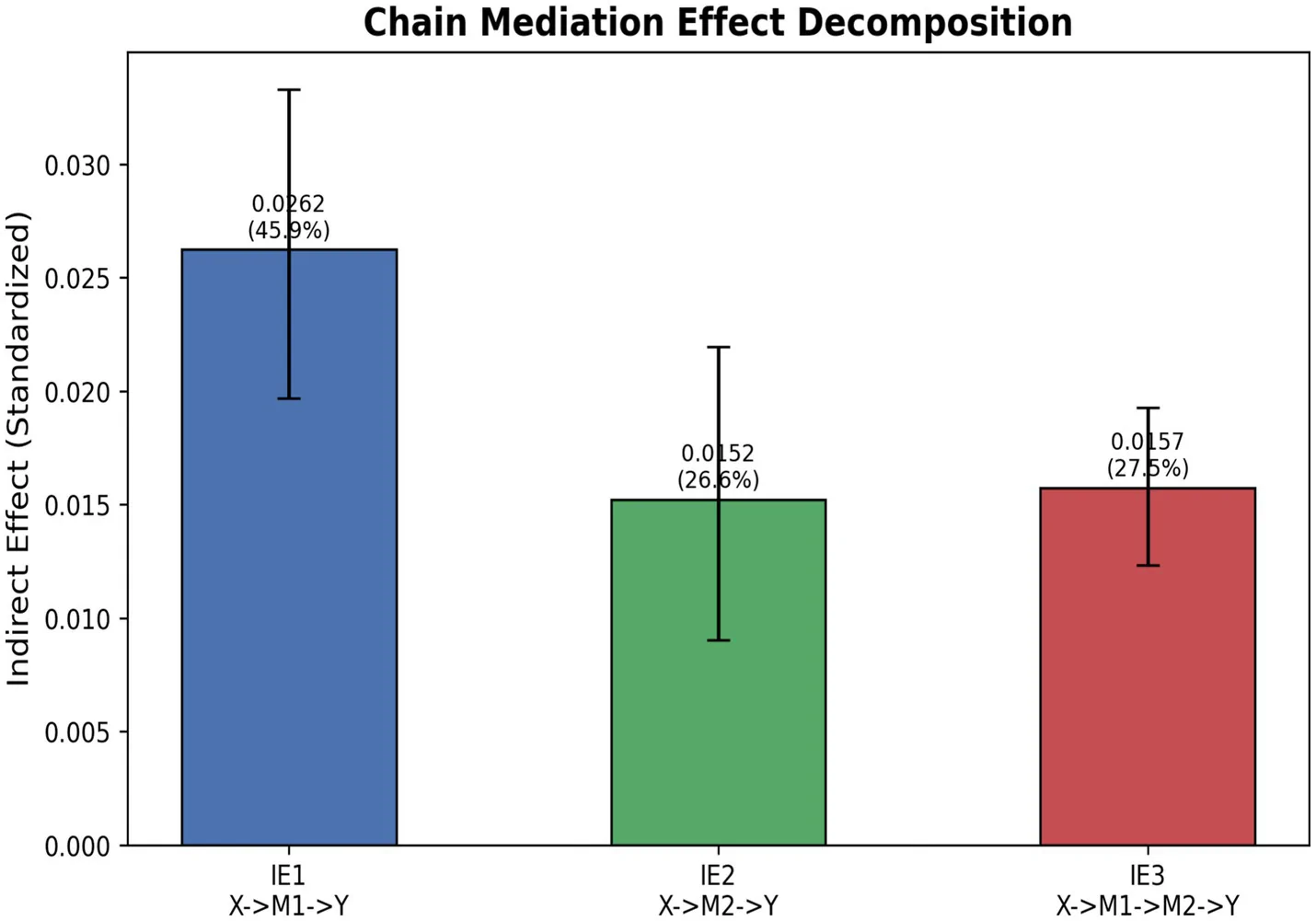
Chain mediating effect decomposition.

### 4.4 Multidimensional heterogeneity analysis

To thoroughly examine the characteristics of the digital health gap across different demographic groups, this study conducted stratified chain-mediating tests across four dimensions: urban/rural settings, gender, age, and educational attainment. Table 4 summarizes the estimated results of chain synergy effects (IE3) for each subsample.

**Table 4:** Comparison of multidimensional heterogeneity chain-specific synergistic effects.

Layered variable	Groups	Sample size N	Chain effect IE3	95% CI	Significance
Area	Urban	2,256	0.0154	[0.0103, 0.0216]	Yes
	Rural	3,077	0.0149	[0.0108, 0.0197]	Yes
Gender	Male	2,398	0.0136	[0.0091, 0.0185]	Yes
	Female	2,935	0.0176	[0.0129, 0.0227]	Yes
Age	18–30	518	0.0012	[−0.023, 0.023]	No
	31–45	1,013	0.0239	[0.011, 0.033]	Yes
	46–60	1763	0.0201	[0.014, 0.028]	Yes
	60+	2039	0.0139	[0.008, 0.018]	Yes
Educational background	Primary	1809	0.0054	[0.002, 0.008]	Yes
	Secondary	2,554	0.0080	[0.004, 0.012]	Yes
	College+	960	0.0036	[−0.005, 0.011]	No

A bootstrap 95% confidence interval that does not include 0 is considered significant.

From an urban–rural perspective, point estimation results indicate that the chain-effect magnitude among urban residents (IE3 = 0.0154) is slightly higher than that among rural residents (IE3 = 0.0148); however, the Bootstrap 95% confidence interval for the difference in intergroup effects includes zero, meaning the intergroup disparity does not reach statistical significance. This finding merely reflects a descriptive trend and does not allow us to conclude that there is a substantive difference in the chain-conduction effect between urban and rural residents.

Along the gender dimension, the point estimate indicates that the chain effect among women (0.0176) is higher than among men (0.0137); however, the intergroup difference – as assessed by a Bootstrap test with a 95% confidence interval of [−0.0106, 0.0031] – includes zero, suggesting that the difference is not statistically significant. Although there is a numerical discrepancy in the point estimates, this descriptive finding may imply that women may have a greater interest in and greater proactive engagement with health-related issues; nonetheless, this hypothesis requires further validation through larger-sample studies.

Along the age dimension, the point estimates exhibit an inverted U-shaped distribution pattern: the IE3 point estimates are relatively higher among the middle-aged groups aged 31–45 years (0.0240) and 46–60 years (0.0219); however, the chain effect within the 1,830-year-old youth group is not statistically significant (IE3 = 0.0012; 95% CI includes 0); for the older adults group aged 60 years and older, the point estimate is 0.0138. The Bootstrap test for the difference in effect sizes between the 31–45 years old group and the group aged 60 years and older yielded a 95% CI of [−0.0021, 0.0252], which includes 0; thus, no statistically significant difference in effect size was observed between these age groups. The analysis of the within-group correlation coefficient for the 18–30 years old group provides only exploratory clues: a weak negative correlation was found between internet health communication and digital health education within this group, suggesting the possibility of an interrupted transmission chain; however, this inference is based solely on descriptive statistics from a subsample and should be interpreted with caution. The relatively low point estimate for the older adults group may serve as an indicative signal of the “silver digital divide,” but the data from this study do not support the existence of a statistically significant difference in intergroup effects.

In terms of educational attainment, the point estimate results exhibit a pattern of higher values in the middle range and lower values at both ends: Secondary school education group (0.0086) > Primary school or below (0.0054) > College degree or above (0.0033; the within-group effect was not significant). The inter-group difference test for the primary school versus college degree or above groups yielded a 95% confidence interval of [−0.0067, 0.0104], which includes zero; thus, the inter-group difference is not statistically significant. Although the chain effect within the low-education subgroup is statistically significant, the point-estimate effect size is relatively small. This descriptive finding suggests that the chain effect within the low-education population may be weakening; however, based on the results of this study, it cannot be concluded that there are substantive differences in the chain effect across different educational attainment groups. The aforementioned stratified point-estimate trend aligns with the direction anticipated by the Digital Health Social Gradient Theory (21), though this alignment is only tentative. Conducting grouped regressions separately for each educational attainment subgroup constitutes a form of heterogeneous stratified analysis; since educational attainment was not included as a covariate in the regression model for each subgroup, this approach does not introduce post-hoc specification bias into the mediation model.

Figure 2 presents a hierarchical comparison of the four-dimensional digital health gap through a forest plot. At the urban–rural level, the urban chain effect (IE3 = 0.0154) is slightly higher than that in rural areas (0.0148), though their confidence intervals overlap significantly; the difference primarily lies in the initial information access stage rather than dissemination efficiency. Regarding gender, women’s value (IE3 = 0.0176) is markedly higher than men’s (0.0137), indicating greater efficiency advantages in health information processing and knowledge translation among females. The age distribution exhibits an inverted U-shape: values are highest for the 31–45 age group (0.0240) and the 46–60 age group (0.0219), approximately twice those observed in the 18–30 age group (0.0012, negative value) and individuals aged 60+ (0.0138), suggesting that middle-aged populations represent the optimal window for digital health communication effectiveness. In terms of educational attainment, secondary education (0.0086) had the highest value, followed by primary education or below (0.0054), while higher education (college level or above, 0.0033; not statistically significant) had the lowest value; the overall effect size was relatively low.

**Figure 2:**
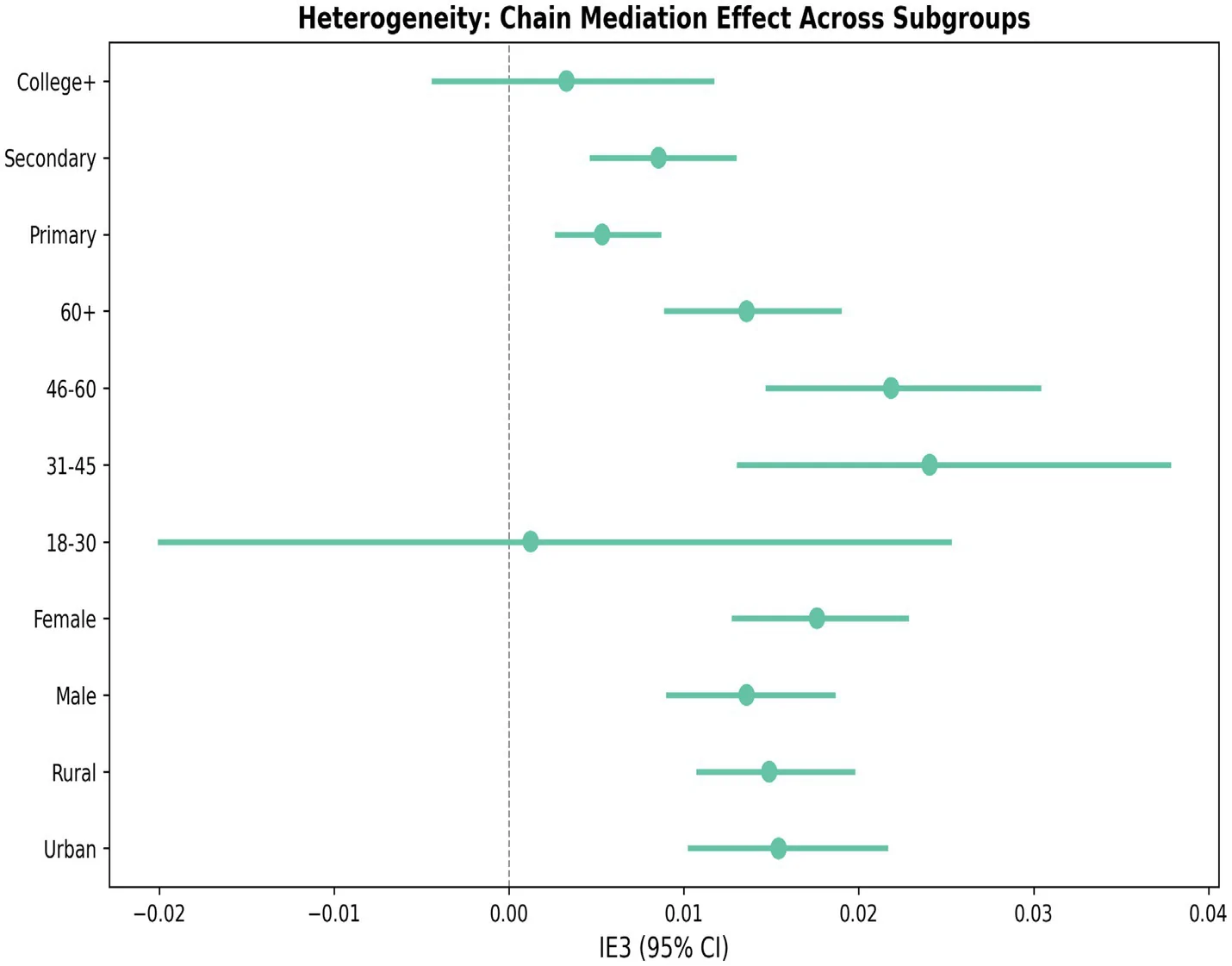
Comparison of multidimensional heterogeneous chain-mediating effects.

### 4.5 Robustness test

To ensure the reliability of the core conclusions, this study conducted four robustness tests. First, all continuous variables were reestimated after Z-score normalization, and the direction and significance of the coefficients in the chain-of-effects path remained unchanged. Second, multicollinearity diagnostics revealed that the VIF values for all independent variables were below 2.1. Third, the benchmark regression consistently employed HC3 heteroscedasticity robust standard errors. Fourth, after IPW inverse probability weighting correction, the coefficient of the core path M2 → Y (b_2_) was 0.204 (*p* < 0.001), highly consistent with the main model. These four tests collectively confirm that the core conclusions remain stable across different model specifications.

Figure 3 presents a forest plot that visually compares the point estimates of IE3 with their 95% confidence intervals under three different model specifications. Model 1 is the baseline OLS model (including only the independent variables); IE3 = 0.0540, 95% CI [0.0457, 0.0622]. Model 2 represents the estimation results after incorporating individual-level control variables; IE3 = 0.0278, 95% CI [0.0230, 0.0329]. Model 3 is the full model after incorporating all control variables; IE3 = 0.0157, 95% CI [0.0123, 0.0193]. The confidence intervals for the three models overlap significantly in the plot, and none of them contain the value 0. As the control variables are progressively added to the model, the magnitude of the IE3 effect gradually declines (from 0.0540 to 0.0157), yet the effect remains statistically significant at all levels, demonstrating the strong robustness of the chain-based synergistic effect to potential confounding factors. The estimated coefficient obtained using IPW inverse probability weighting (b₂ = 0.204, *p* < 0.001) is consistent with the direction observed in the main model.

**Figure 3:**
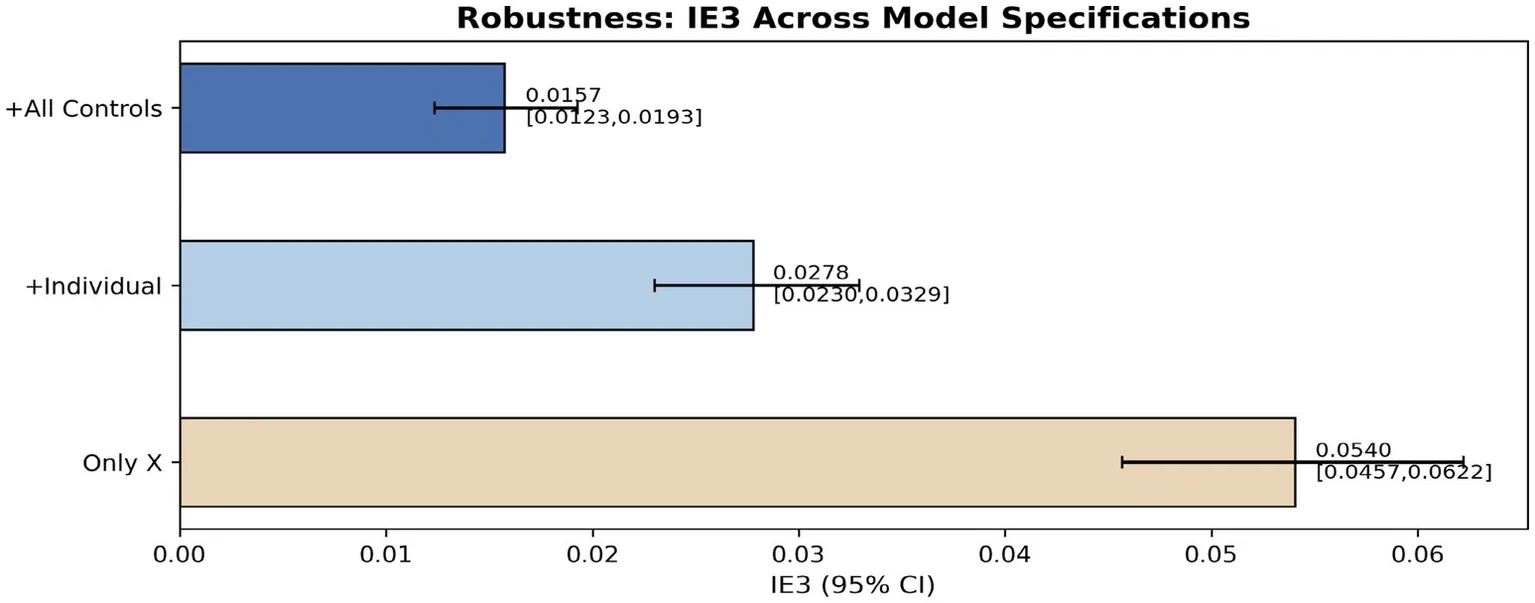
Robustness test forest plot.

## 5 Discussion

### 5.1 Theoretical interpretation of the core findings

The core empirical findings of this study can be summarized into three progressive levels.

First, a significant positive statistical overall association was observed between online health communication and community-based health promotion (*β* = 0.0855, *p* < 0.001); this statistical pattern aligns with the core theoretical expectations of the Human-Machine Collaborative Health Ecology Theory (2). Cohen’s *f*^2^ = 0.1253 falls between small (0.02) and moderate (0.15) effects. Even after controlling for all variables, this independent effect remains statistically significant, demonstrating that internet-driven health information dissemination transcends individual socioeconomic disparities and yields health-promoting effects across broad population groups.

Second, health literacy demonstrated a significant independent mediating role (IE1 = 0.0262, accounting for 45.9% of the indirect effect), confirming the pivotal role of the “cognitive filtering” mechanism in information-to-behavior translation. This finding aligns with the conclusions of Berkman et al. (18) and Wei and Jia (10), further solidifying health literacy’s central mediating position in digital health communication. Additionally, Nutbeam’s hierarchical model of health literacy (functional literacy → interactive literacy → critical literacy) provides a crucial theoretical framework for understanding this mechanism (26).

Third—and this represents the core theoretical contribution of the present study—the chain-like synergistic path (IE3 = 0.0157) is statistically significant, indicating that, based on cross-sectional data, an observed chain-like statistical association pattern—namely “Internet dissemination → digital literacy → education → behavior” —exists among the variables; this pattern suggests that these three elements may theoretically form a “progressive synergy” gain feedback loop; however, the cross-sectional data are insufficient to establish a causal time-series relationship. The underlying mechanism of this synergistic effect can be explained from a cognitive psychology perspective: fragmented knowledge provided by internet dissemination triggers health awareness with low cognitive load; health literacy enables information filtering through critical evaluation; while systematic online learning integrates scattered knowledge points into structured frameworks, facilitating deep knowledge encoding and long-term memory retention via repetitive, phased learning activities. The cross-theoretical model proposed by Prochaska and Velicer (22) also corroborates this sequential transformation pathway. All three components are indispensable; any deficiency in any stage significantly diminishes the overall effectiveness of health promotion efforts.

### 5.2 Dialogue with existing literature and theoretical contributions

This study engages in substantive dialogue with existing literature across multiple dimensions. First, unlike the convenience-sample-based experimental studies of Aggarwal et al. (3) and Kilic et al. (1), our research employs the CGSS nationally stratified representative sample (*N* = 5,333) to validate the robustness of the chain mechanism across macro populations, providing cross-sample evidence for the external validity of the human-machine collaborative health ecology theory. Second, adhering to the methodological framework of serial mediation proposed by Hayes (25) and Zhao et al. (16), we systematically examine the complete causal chain— “information acquisition → cognitive filtering → systematic learning → behavioral transformation” —within a single model, demonstrating that health literacy and digital health education exhibit a clear sequential relationship rather than an interchangeable substitute relationship. Third, two findings regarding study heterogeneity warrant particular discussion: First, the chain effect among the 18–30-year-old youth group did not reach statistical significance; internal mechanism analysis reveals a weak negative correlation between internet usage and digital health education among this demographic, suggesting a generational shift in information acquisition patterns—where young individuals primarily obtain health information through fragmented channels such as social media platforms and short videos rather than participating in structured online courses. Secondly, the chain effect in the group with a college degree or higher was not significant; combined with the fact that the direct correlation coefficient between internet usage and health promotion in this group was only 0.028, this suggests that health behaviors among highly educated individuals may rely more heavily on non-internet channels such as professional medical consultation and fitness guidance, indicating limited marginal contribution of the chain pathway. These findings align with Neter and Brainin’s (21) classic argument regarding the digital divide in e-health literacy, as well as Paasche-Orlow and Wolf’s (20) theoretical framework on the causal pathways of health literacy. The WHO Global Framework for Digital Health Literacy (27) provides an international reference framework for policy translation in this study.

### 5.3 Policy implications

Building on findings from existing specialized intervention studies, the SPOC model is characterized by its small-scale, highly interactive nature, making it particularly well-suited for health education settings targeting general community residents (12). It should be noted that, since the proxy variables used in this study from the CGSS cannot directly measure residents’ actual participation in MOOCs or SPOC programs, this policy insight is an extended reference drawn from the intervention literature and does not represent a direct statistical conclusion derived from this chain-mediation model. The study by Li et al. (12) demonstrated that the intervention group receiving SPOC-based interventions combined with simulated training outperformed the control group—which received only SPOC-based interventions—significantly across all dimensions of health competence; this finding suggests that digital health education targeting rural populations or individuals with lower educational attainment should be complemented with offline practical coaching sessions to facilitate the deep internalization of knowledge and the transformation of behaviors.

Based on the statistical correlation patterns derived from cross-sectional observations and in conjunction with existing theoretical literature, this study proposes the following exploratory policy recommendations; it should be emphasized that cross-sectional data cannot establish causal relationships; therefore, the actual effectiveness of policy implementation requires further validation through longitudinal tracking or field intervention studies.

First, establish a tripartite collaborative closed-loop system comprising “internet traffic diversion → literacy training → course learning.” Currently, digital public health practices across regions remain fragmented, with AI tool promotion, digital literacy training, and online course development being pursued separately by different departments without systematic integration (15). In regions with adequate conditions, pilot cross-departmental coordination mechanisms to achieve data interoperability and operational synergy among AI-based health Q&A tools, community digital literacy workshops, and online course platforms.

Second, implement a tiered health literacy intervention program. It should be noted that the difference in the inter-group chain-effect was not statistically significant; these tiered policy recommendations are primarily derived from the current reality of digital health stratification across urban and rural areas as well as across different educational backgrounds in China, as well as from existing literature on the digital divide, while also taking into account the descriptive point-estimation trend observed in this study. For rural populations and individuals with lower educational attainment, it is recommended to provide complementary offline hands-on training sessions on operating digital devices in community settings, alongside simplified audio-based online courses, in order to lower the participation barrier at the cognitive mediation stage (12); for middle-aged populations, online micro-courses on AI critical thinking should be offered, focusing on identifying algorithmic bias and discerning the authenticity of information.

Third, bridge the gap in digital health infrastructure between urban and rural areas by designating primary healthcare clinics and community service centers as fixed endpoints for digital health education. Wu et al. emphasize that China’s digital health policy should achieve a strategic balance between technological innovation and equitable access (15); this study provides a concrete empirical pathway for implementing a tiered intervention approach in this regard.

### 5.4 Research limitations and future directions

This study has the following limitations.

First, cross-sectional data cannot definitively establish the causal direction, the mediation sequence is non-identifiable, and the direct effect c’ is sensitive to the IPW weighting specification; therefore, causal inference should be conducted with caution. Additionally, the chain mediation approach employed in this study only examines the unidirectional transmission sequence pre-specified by the researchers; cross-sectional data are insufficient to test bidirectional feedback or mutually reinforcing cyclical processes among variables. The notion of a “synergistic gain closed-loop” is merely a theoretical hypothesis and has not been empirically confirmed by the data collected in this study.

Second, all core variables in this study are proxy indicators, which entails the limitation of an incomplete alignment between theoretical constructs and observed measurement indicators. The CGSS-2023 is a comprehensive social survey and does not include any specialized scales tailored to the digital health domain. M1—which includes measures of Mandarin proficiency, educational attainment, and reading habits—can capture the underlying dimensions of health literacy, such as linguistic comprehension, basic cognitive abilities, and proactive information seeking; however, it lacks higher-order, specialized dimensions such as critical evaluation of health information or algorithm literacy. M2— “leisure learning and self-education” —can reflect an individual’s behavioral tendency toward systematic learning; nonetheless, it cannot distinguish whether the learning topic pertains to health, whether it occurs via online or offline channels, or the specific mode of delivery. The independent variable X only measures the overall intensity of internet usage and cannot differentiate whether participants actually encounter health-related information or AI-generated content; the dependent variable Y uses physical exercise alone to represent health promotion, thereby failing to encompass a broader range of health-promoting behaviors. In short, this study’s model examines a chain-of-pathways structure constructed from proxy variables and then extrapolates this framework to the theoretical mechanisms underlying online health communication; however, it does not directly assess the actual impact of ordinary individuals using AI-based health tools or participating in MOOC/SPOC health-related courses. Since M2 and Y are measured using single-item items, it is not possible to calculate the internal consistency reliability; furthermore, the EFA conducted for M1 yielded a single-factor variance explanation of 56.4% (with a communality of 43.3%), suggesting that specialized scales could be employed in future studies to further refine the measurement approach.

Third, and representing the core theoretical contribution of this study, the chain-shaped collaborative path (IE3 = 0.0157) is statistically significant, indicating that, based on cross-sectional data, an observed chain-shaped statistical association pattern—namely “Internet dissemination → digital literacy → education → behavior” —exists among the variables. This pattern suggests a potential theoretical logic of progressive synergy among these three components, which could give rise to a gain-based feedback loop; however, cross-sectional data are insufficient to confirm the causal temporal sequence of the variables over time, nor can they substantiate the existence of a bidirectional, mutually reinforcing cyclical process.

Fourth, both the core chain effect (IE3 = 0.0157) and the total effect (*β* = 0.0855) are relatively small; the model’s *R*^2^ = 0.1801, indicating that the practical magnitude of these effects should be taken into account when translating policy interventions into practice. There are minimal rounding differences between the coefficients obtained from different statistical software packages (OLS and PROCESS); therefore, the study prioritizes reporting the raw total effect (*β* = 0.0855) derived from the PROCESS chain mediation model in the main text.

Fifth, the differences across heterogeneous groups were not statistically significant; the analysis of educational stratification was limited by the small sample sizes within each group. Future research could employ panel data, specialized AI-based measurement scales, and Confirmatory Factor Analysis (CFA) to further test the robustness and external validity of these findings.

Sixth, this study integrates educational attainment into the construction of the M1 mediation model rather than treating education as an independent control variable; while this approach mitigates post-hoc bias, it fails to isolate the direct effect of education itself—representing a trade-off inherent in using secondary data for mediation modeling.

Seventh, in the multidimensional heterogeneity analysis conducted in this study, although the chain-mediated moderation effects within certain subgroups were statistically significant, the Bootstrap tests for the overall between-group effect differences did not reach statistical significance; thus, the stratified results merely provide descriptive point-estimation trends and cannot serve as conclusive evidence of substantive differences in between-group effects.

Eighth, while the CGSS employs a stratified, multi-stage complex probability sampling design, the limitations imposed by the variables in the publicly available dataset—and the fact that the PROCESS tool does not support the Bootstrap method for complex survey chain mediation—mean that the primary analysis in this study did not incorporate adjustments for sampling weights, clustering, or stratification. This study uses IPW (Inverse Probability Weighting) as an alternative robustness check; however, IPW is not entirely equivalent to the official survey sampling weights. As a result, the standard errors of the parameter estimates and the magnitude of the effects may differ somewhat from those obtained when the complex sampling design is fully accounted for.

## 6 Conclusion

This study utilizes the National Stratified Representative Micro-Database of Residents (*N* = 5,333) from the 2023 China General Social Survey (CGSS), constructs proxy indicators based on existing questionnaire items, and employs the Hayes PROCESS Model 6 serial chain-mediated model to examine the chain-conduction effect. Based on these findings, the study infers a progressive synergistic gain mechanism through which internet-based health communication, health literacy, and digital health education contribute to health promotion among residents. The following key conclusions are drawn:

First, cross-sectional data reveal a significant positive overall statistical association between online health communication and community health promotion (*β* = 0.0855, *p* < 0.001); this association is primarily mediated through intermediate variables (the direct effect is not significant in OLS – the 95% CI includes 0; however, the direct effect is significant after IPW weighting, so caution should be exercised when interpreting the full mediation effect). This statistical pattern suggests, from a theoretical perspective, that an indirect transmission pathway of “cognitive screening → systematic learning” may underlie the relationship between online health communication and health behaviors. Relying solely on AI-driven information push strategies without simultaneously establishing a framework for fostering cognitive literacy and developing an online education system will fundamentally limit the effectiveness of health promotion efforts.

Second, the chain-mediated moderation decomposition identified three statistically significant indirect mediation pathways; the cross-sectional observational data reveal a chain-like statistical pattern that aligns with the theoretical model “Information Input → Cognitive Filtering → System Internalization → Behavioral Transformation.” Among these, the single health literacy pathway (IE1 = 0.0262, 45.9%) accounts for the largest share, confirming that health literacy serves as the primary mediating factor in the transformation of information-based behaviors as a “cognitive barrier”; the single digital health education pathway (IE2 = 0.0152, 26.6%) demonstrates the independent driving effect of systematic learning on behavioral transformation; and the core chain-mediated synergistic pathway (IE3 = 0.0157, 27.5%) is statistically significant, suggesting at the theoretical level that these three pathways exhibit a progressive synergistic relationship and have the potential to form a gain-based feedback loop. It is important to note that this study utilizes cross-sectional data, which can only test pre-specified unidirectional chain-like statistical associations; it cannot confirm whether this sequence actually unfolds over time, nor can it verify the existence of a bidirectional, mutually reinforcing cyclical effect. The Bootstrap confidence intervals for all three pathways do not include 0, providing statistical support for the three research hypotheses (H1, H2, H3) at the pattern-level.

Third, the multidimensional heterogeneity analysis reveals the complex structure of the digital health gap, providing guidance for policy differentiation in this study. At the urban–rural dimension, the chain effect among urban residents (0.0154) was slightly higher than that among rural residents (0.0148; the intergroup difference was not statistically significant). At the gender dimension, the chain effect was slightly higher among women (0.0176) than among men (0.0137; the intergroup difference was not statistically significant). At the age dimension, the strongest effect was observed among the middle-aged population aged 31–60 (0.0240 for the 31–45 age group and approximately 0.0219 for the 46–60 age group), exhibiting a significant “inverted U-shaped” distribution pattern. At the education level dimension, the chain effect was extremely weak among individuals with low educational attainment (primary school: 0.0054; secondary school: 0.0086); the effect among individuals with an associate degree or higher was not statistically significant (0.0033). Individuals with higher educational attainment have more diverse sources of health information (e.g., fitness guidance, medical consultations, etc.), and the chain pathway is not their primary means of health promotion. Although the differences between groups were not statistically significant, the trends observed in the point estimates are consistent with theoretical expectations, suggesting that the digital health gap may result from the cumulative effect of multiple structural factors—not only the “accessibility gap” (the disparity in digital infrastructure between urban and rural areas) but also the combined effects of the “literacy gap” (differences in information discernment ability) and the “assimilation gap” (differences in knowledge translation efficiency). From the descriptive trends in the point estimates, the point estimate values for the chain effect among rural residents and individuals with low educational attainment are relatively small, indicating a potential risk of weakening in the transmission chain within these groups; however, the difference in the chain effect across the groups in this study did not pass the Bootstrap statistical significance test. Given the current digital health disparity landscape, rural populations and individuals with lower educational attainment remain key groups that warrant prioritized attention in the development of digital public health education initiatives under the “Healthy China” initiative.

Fourth, four robustness tests—including IPW inverse probability weighting, standardized reestimation, VIF diagnosis, and HC3 robust standard error—consistently confirm that the core conclusions remain stable across different model specifications and estimation methods, demonstrating strong empirical reliability.

In summary, this study integrates the theories of human-machine collaborative health ecology, digital health literacy, and lifelong online education at the theoretical level, establishing a localized empirical analysis framework tailored to China’s social stratification structure; at the empirical level, it examines the serial chain-based collaborative mechanism of “internet dissemination → literacy → education → behavior” using nationally representative samples; and at the policy level, it proposes a “trinity” collaborative closed-loop system along with its hierarchical implementation pathways. The research findings provide quantitative empirical support and precise stratified intervention guidelines for reconstructing the digital public health education system and narrowing health disparities across the population under the “Healthy China 2030” strategic framework. Future studies can leverage long-term panel data, specialized AI survey instruments, and mixed-method approaches to further refine the localized theoretical framework for digital public health education.

## Data Availability

The original contributions presented in the study are included in the article/supplementary material, further inquiries can be directed to the corresponding author.

## References

[1] Kilic K Weck S Kampik T Lindgren H. Argument-based human-AI collaboration for supporting behavior change to improve health. Front Artif Intell. (2023) 6:1069455. doi: 10.3389/frai.2023.1069455, 36872933 PMC9979214

[2] Romero-Rodriguez LM Castillo-Abdul B. From apomediation to AImediation: generative AI and the reconfiguration of informational authority in health communication. J Prim Care Community Health. (2025) 16:21501319251381878. doi: 10.1177/21501319251381878, 40990570 PMC12461036

[3] Aggarwal A Tam CC Wu D Li X Qiao S. Artificial intelligence-based chatbots for promoting health behavioral changes: systematic review. J Med Internet Res. (2023) 25:e40789. doi: 10.2196/40789, 36826990 PMC10007007

[4] Singh B Olds T Brinsley J Dumuid D Virgara R Matricciani L . Systematic review and meta-analysis of the effectiveness of chatbots on lifestyle behaviours. NPJ Digital Medicine. (2023) 6:118. doi: 10.1038/S41746-023-00856-1, 37353578 PMC10290125

[5] Eysenbach G. Medicine 2.0: social networking, collaboration, participation, apomediation, and openness. J Med Internet Res. (2008) 10:e22. doi: 10.2196/jmir.1030, 18725354 PMC2626430

[6] Norman CD Skinner HA. eHEALS: the eHealth literacy scale. J Med Internet Res. (2006) 8:e27. doi: 10.2196/jmir.8.4.e27, 17213046 PMC1794004

[7] Sørensen K Van den Broucke S Fullam J Doyle G Pelikan J Slonska Z . Health literacy and public health: a systematic review and integration of definitions and models. BMC Public Health. (2012) 12:80. doi: 10.1186/1471-2458-12-80, 22276600 PMC3292515

[8] Sørensen K Pelikan JM Röthlin F Ganahl K Slonska Z Doyle G . Health literacy in Europe: comparative results of the European health literacy survey (HLS-EU). Eur J Pub Health. (2015) 25:1053–8. doi: 10.1093/eurpub/ckv043 PMC4668324 25843827

[9] Stellefson M Hanik B Chaney B Chaney D Tennant B Chavarria EA. eHealth literacy among college students: a systematic review with implications for eHealth education. J Med Internet Res. (2011) 13:e102. doi: 10.2196/jmir.1703, 22155629 PMC3278088

[10] Wei P Jia H. Can digital health literacy promote residents' physical and mental health? An empirical analysis based on Chinese general social survey. Front Public Health. (2025) 13:1654330. doi: 10.3389/fpubh.2025.1654330, 41179787 PMC12575218

[11] Mackert M Mabry-Flynn A Champlin S Donovan EE Pounders K. Health literacy and health information technology adoption: the potential for a new digital divide. J Med Internet Res. (2016) 18:e264. doi: 10.2196/jmir.6349, 27702738 PMC5069402

[12] Li H Zhao W Li B Li Y. Effects of the small private online course combined with simulation-based training in a patient safety education program among nursing students: a quasi-experimental study. Int J Nurs Sci. (2023) 10:555–61. doi: 10.1016/J.IJNSS.2023.09.014, 38020829 PMC10667291

[13] Luo A Yu Z Liu F Xie W. The chain mediating effect of the public online health information-seeking behavior on doctor-patient interaction. Front Public Health. (2022) 10:874495. doi: 10.3389/fpubh.2022.874495 PMC9201044 35719674

[14] Chesser A Burke A Reyes J Rohrberg T. Navigating the digital divide: a systematic review of eHealth literacy in underserved populations in the United States. Inform Health Soc Care. (2016) 41:1–19. doi: 10.3109/17538157.2014.948171, 25710808

[15] Wu G Ning H Yuan Y Chen J Li G Liu S . Topic identification and content analysis of internet medical policies under the background of healthy China 2030. Health Res Policy Syst. (2024) 22:132. doi: 10.1186/S12961-024-01226-3, 39350159 PMC11440693

[16] Zhao X Lynch JG Chen Q. Reconsidering baron and Kenny: myths and truths about mediation analysis. J Consum Res. (2010) 37:197–206. doi: 10.1086/651257

[17] Bickmore TW Silliman RA Nelson K Cheng DM Winter M Henault L . A randomized controlled trial of an automated exercise coach for older adults. J Am Geriatr Soc. (2013) 61:1676–83. doi: 10.1111/jgs.12449, 24001030

[18] Berkman ND Sheridan SL Donahue KE Halpern DJ Crotty K. Low health literacy and health outcomes: an updated systematic review. Ann Intern Med. (2011) 155:97–107. doi: 10.7326/0003-4819-155-2-201107190-00005, 21768583

[19] Bandura A. Health promotion by social cognitive means. Health Educ Behav. (2004) 31:143–64. doi: 10.1177/1090198104263660, 15090118

[20] Paasche-Orlow MK Wolf MS. The causal pathways linking health literacy to health outcomes. Am J Health Behav. (2007) 31:S19–26. doi: 10.5555/ajhb.2007.31.supp.S19, 17931132

[21] Neter E Brainin E. eHealth literacy: extending the digital divide to the realm of health information. J Med Internet Res. (2012) 14:e19. doi: 10.2196/jmir.1619, 22357448 PMC3374546

[22] Prochaska JO Velicer WF. The transtheoretical model of health behavior change. Am J Health Promot. (1997) 12:38–48. doi: 10.4278/0890-1171-12.1.38 10170434

[23] van der Vaart R Drossaert C. Development of the digital health literacy instrument: examining the factor structure of the eHealth literacy scale. J Med Internet Res. (2017) 19:e27. doi: 10.2196/jmir.6709 PMC5358017 28119275

[24] Tudor-Locke C Craig CL Brown WJ Clemes SA De Cocker K Giles-Corti B . How many steps/day are enough? For adults. Int J Behav Nutr Phys Act. (2011) 8:79. doi: 10.1186/1479-5868-8-79, 21798015 PMC3197470

[25] Hayes AF. Introduction to Mediation, Moderation, and Conditional process Analysis. New York, NY: Guilford Press (2022).

[26] Nutbeam D. The evolving concept of health literacy. Soc Sci Med. (2008) 67:2072–8. doi: 10.1016/j.socscimed.2008.09.050, 18952344

[27] WHO. Global Strategy on digital Health 2020–2025. Geneva: World Health Organization (2021).

